# The Mediating Effect of the Choline-to-Betaine Ratio on the Association Between *PEMT* rs7946 and Digestive System Cancer: A Nested Case–Control Study in a Chinese Population

**DOI:** 10.1016/j.cdnut.2024.102075

**Published:** 2024-01-04

**Authors:** Qiangqiang He, Yaping Wei, Hehao Zhu, Yun Song, Ping Chen, Binyan Wang, Hanping Shi, Peiwu Qin

**Affiliations:** 1 Shenzhen International Graduate School, Tsinghua University, Shenzhen, China; 2 Shenzhen Evergreen Medical Institute, Shenzhen, China; 3 College of Public Health, Shanghai University of Medicine and Health Sciences, Shanghai, China; 4 School of Science, China Pharmaceutical University, Nanjing, China; 5 College of Pharmacy, Jinan University, Guangzhou, China; 6 Inspection and Testing Center, Key Laboratory of Cancer FSMP for State Market Regulation, Shenzhen, China; 7 Institute of Biomedicine, Anhui Medical University, Hefei, China; 8 Department of Gastrointestinal Surgery/Department of Clinical Nutrition, Beijing Shijitan Hospital, Capital Medical University, Haidian District, Beijing, China; 9 Beijing International Science and Technology Cooperation Base for Cancer Metabolism and Nutrition, Haidian District, Beijing, China; 10 Key Laboratory of Cancer FSMP for State Market Regulation, Haidian District, Beijing, China; 11 Center of Precision Medicine and Healthcare, Tsinghua-Berkeley Shenzhen Institute, Shenzhen, China; 12 Institute of Biopharmaceutics and Health Engineering, Tsinghua Shenzhen International Graduate School, Shenzhen, China

**Keywords:** 1-carbon metabolism, phosphatidylethanolamine N-methyltransferase, gene polymorphism, digestive system cancer, case–control study

## Abstract

**Background:**

The enzyme phosphatidylethanolamine N-methyltransferase (*PEMT*) is responsible for synthesizing phosphatidylcholine by methylating phosphatidylethanolamine. We hypothesized that a polymorphism of the **PEMT** gene, rs7946, is involved in carcinogenesis.

**Objectives:**

We aimed to investigate the relationship between *PEMT* rs7946 and digestive system cancer and examine possible effect modifiers and mediators.

**Methods:**

We conducted a nested, case–control study within the China H-type Hypertension Registry Study, including 751 cases and 1:1 matched controls. To assess the association of *PEMT* rs7946 and digestive system cancer, we estimated odds ratios with 95% confidence intervals (CIs) using conditional logistic regression. We used the bootstrap test to examine the potential mediating effects of related metabolites.

**Results:**

Our results revealed that wild-type homozygous CC genotype carriers of *PEMT* rs7946 had a significantly increased risk [odds ratio (OR): 1.31; 95% CI: 1.04, 1.66; *P* = 0.023] compared with the TT/CT combined genotypes. The effect was found to be more pronounced in individuals with a lower choline-to-betaine ratio (<0.412, *P*-interaction = 0.021). Furthermore, the mediation analysis indicated that the choline-to-betaine ratio played a significant role in mediating 13.55% of the association between *PEMT* rs7946 and digestive system cancer (*P* = 0.018).

**Conclusions:**

Our study suggested that *PEMT* rs7946 may affect risk of digestive system cancer through direct and indirect pathways, and the choline-to-betaine ratio may partially mediate the indirect effect.

This trial was registered at Chinese Clinical Trial Registry as ChiCTR1800017274.

## Introduction

Worldwide, an estimated 19.3 million new cancer cases and almost 10.0 million cancer deaths occurred in 2020 [1]. In China, there were 4,568,756 incidences and 3,002,899 deaths from cancer [2], among these cases, cancers affecting the digestive system, including but not limited to those in the stomach, liver, rectum, and esophagus region, are notable for their aggressiveness and high mortality rates, contributing significantly to the overall burden of cancer. The poor prognosis of digestive system cancer is mainly due to a lack of timely and accurate diagnosis. Therefore, exploring novel biomarkers from multiple dimensions might be helpful for early diagnosis and prevention.

The etiologies of cancer are complex and not well understood yet. They are most likely caused by genetic and environmental susceptibility factors. The importance of 1-carbon metabolism (OCM) in cancer was initially recognized over 70 y ago. It is supposed that disturbances in OCM may potentially facilitate carcinogenesis by causing aberrant DNA synthesis and DNA methylation. Thus, the roles of components involved in the OCM pathway in carcinogenesis have been of intense interest for decades [3]. Genetic variations in OCM genes were also reported to influence the functions in 1-carbon supply and modify cancer risk, previous studies have investigated and depicted their associations with risk of multiple cancers [[4], [5], [6]].

Choline is an alternative source of methyl groups for cellular methylation reactions and is involved in OCM through its sole oxidative product, betaine. The aberrant status of choline metabolism has been linked to malignant transformation [7]. Phosphatidylethanolamine N-methyltransferase (*PEMT*, EC 2.1.1.17), a choline-related gene, codes an enzyme that catalyzes the only reaction for de novo synthesis of choline in the body via methylation of phosphatidylethanolamine to form phosphatidylcholine (PC), using S-adenosyl methionine as the methyl donor [8]. Under normal conditions, the *PEMT* pathway accounts for 20%–30% of PC synthesized [9], and a considerable amount of choline can be produced de novo from PC [10].

The *PEMT* gene is highly polymorphic [11]. A functional polymorphism of the *PEMT* gene, rs7946, has been reported to be associated with breast cancer risk, and significant interactions were observed between choline intake [12] and betaine intake [13]. In the present study, we classified cancer cases coded as C15–C26 in the International Classification of Diseases, 10th Revision (ICD-10), under the comprehensive category of digestive system cancers (Supplemental Table 1), which represent the most frequently occurring cancers in the Chinese population [14,15]. However, limited data currently exist to demonstrate the potential associations between this variant and digestive system cancer. To address these knowledge gaps, we conducted a nested, case–control (NCC) study within a large, community-based, prospective cohort study in China. We aimed to investigate the potential association between *PEMT* rs7946 and digestive system cancer risk and to explore possible effect modifiers and potential mediating effects from choline-related metabolites.

## Methods

### Study design and population

An NCC study was conducted within the China H-type Hypertension Registry Study (CHHRS), the methods and primary outcomes of the CHHRS have been reported elsewhere [16]. Briefly, the CHHRS project is a community-based, prospective, observational, real-world registry study conducted in Lianyungang, Jiangsu Province, and Rongcheng, Shandong Province, China. Started in 2018, it was designed to investigate the prevalence of H-type hypertension and related risk factors in China. There were no prespecified exclusion criteria, except for those who were unable to participate in the follow-up or who were unable to demonstrate informed consent according to the study protocol. Eligible persons were those aged ≥18 y with essential hypertension, defined as seated systolic blood pressure of ≥140 mm Hg and/or seated diastolic blood pressure of ≥90 mm Hg at the screening visit. The study was conducted in accordance with the tenets of the Declaration of Helsinki and approved by the Ethics Committee of the Institute of Biomedicine, Anhui Medical University, Hefei, China. Informed consent was obtained from all subjects involved in the study.

### Primary outcome and case ascertainment

The primary outcome of interest in this study was incident digestive system cancer during the follow-up period from 2018 to 2019, excluding any previous history of cancer or tumor as assessed at the baseline study. As described previously [16], information on newly diagnosed cancer for all participants was obtained from the China Center for Disease Control at 2 centers and crossverified through the national health insurance system that was electronically linked to all hospitalization data and participants’ self-report during the follow-up visits every 3 mo as part of CHHRS. All reported cancers were coded according to the ICD-10.

### Selection of matched case–control pairs

**FIGURE 1:**
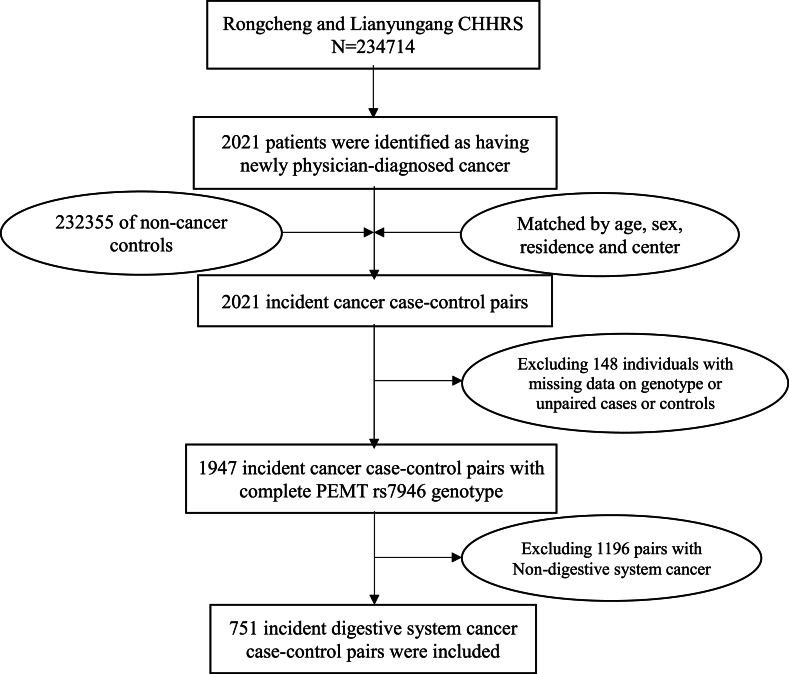
Flowchart of study participants in the nested case–control study within the CHHRS. Abbreviations: CHHRS, China H-type Hypertension Registry Study; *PEMT*, phosphatidylethanolamine N-methyltransferase.

As shown in Figure 1, during the follow-up period, 2021 patients were identified to have new physician-diagnosed cancer. Then, 232,355 participants who were still alive and never had cancer before the study and during the follow-up period were matched with incident cancer cases in a 1:1 ratio. The matching factors included age (±1 y), sex, residence, and center at baseline. After excluding participants with missing data on genotype or unpaired cases or controls (*n* = 148), we obtained 1947 cancer case–control pairs. A total of 1196 pairs of subjects with nondigestive system cancer were excluded; finally, 751 incident digestive system cancer case–control pairs with complete *PEMT* rs7946 genotype were selected for the final data analysis.

### *PEMT* rs7946 genotyping

Genomic DNA was extracted from leukocytes of venous blood by standard procedures using MagicPure Blood Genomic DNA kits (TransGen Biotech), and the concentration was adjusted to 50–60 ng/mL. Genotyping was performed using the DR MassARRAY system (Agena Bioscience). All primers were designed using the software MassARRAY Assay Design Suite, version 2.2 (Agena Bioscience). DNA samples were amplified by multiplex polymerase chain reactions, after which, the polymerase chain reaction products were used for locus-specific single-base extension reactions. The resulting products were desalted and subsequently transferred to a 96-well SpectroCHIP bioarrary (Agena Bioscience) using a DR MassARRAY Nanodispenser RS1000 (Agena Bioscience). Allele detection was performed using matrix-assisted laser desorption ionization–time of flight (Agena BioScience) mass spectrometry, and the data were analyzed using a software MassARRAY Typer Analyzer, version 4.1 (Agena Bioscience), following the manufacturer’s standard protocols.

The entire genotyping process was performed with no knowledge of participant case–control status or clinical data. In addition, 10% of the samples were randomly selected for repeated testing, and the results were found to be 100% consistent.

### Statistical analysis

Normally distributed variables were presented as means ± SDs and compared using paired *t* tests. Nonnormally distributed variables were presented as median (IQR) and analyzed using rank-sum tests. Categorical variables were presented as *n* (%) and compared using the χ^2^ test. The characteristics of T allele carriers and CC genotypes were compared using the Mann–Whitney *U* test.

We applied both conditional and unconditional models based on the design of our matched case–control study for digestive system cancer. Specifically, we used conditional logistic regression models to assess the association between variant *PEMT* rs7946 and incident digestive system cancer. In addition, we conducted a subgroup analysis using unconditional logistic regression models to evaluate potential effect modification by certain important variables related to the outcome, namely, digestive system cancer.

A mediation analysis was performed, using variant *PEMT* rs7946 as the independent variable, digestive system cancer as the dependent variable, and the choline-to-betaine ratio as the mediator. To analyze the mediation model, we employed the nonparametric bootstrapping method and utilized the mediate function in the mediation package (package version: 4.5.0). For an accurate assessment of the mediation effect, we conducted a bootstrapping procedure using 1000 bootstrap samples. Additionally, to address potential confounding factors, we incorporated 5 covariates into the model: *1*) smoking status, *2*) alcohol drinking status, *3*) homocysteine concentrations, *4*) vitamin B-12 concentration, and *5*) total folate concentrations.

A 2-tailed *P* < 0.05 was considered statistically significant in all analyses. All statistical analyses were performed using R software, version 4.0.5 (http://www.R-project.org/).

## Results

### Baseline characteristics of the participants

The baseline characteristics of 751 incident digestive system cancer cases and matched controls were summarized in Table 1. Most of the variables showed no significant difference between cases and controls, including all of the selected clinical measures and several serum concentrations for components of OCM. However, compared with matched controls, the cases were more likely to have higher serum betaine concentrations, although this difference was just above the margin of significance (*P* = 0.054) and had a significantly lower value of choline-to-betaine ratio (*P* = 0.033). In addition, the proportion of current smokers was higher but that of who have never smoked was lower in the cases. This difference showed statistical significance (*P* = 0.020).

**TABLE 1:** Baseline characteristics of the study participants by case–control status (pairs = 751)^1^

Baseline variables	Total (*N* = 1502)	Cases (*n* = 751)	Controls (*n* = 751)	*P* value
Age (y)	69.516 ± 7.540	69.52 ± 7.55	69.51 ± 7.54	0.995
Male	522 (34.8)	261 (34.8)	261 (34.8)	1.000
Education				0.956
Primary school or lower	1018 (67.8)	510 (50.1)	508 (49.9)	
Secondary and above	484 (32.2)	241 (32.1)	243 (32.4)	
Smoking status				0.020
Current	438 (29.2)	243 (32.4)	195 (26.0)	
Former	180 (12.0)	90 (12.0)	90 (12.0)	
Never	884 (58.9)	418 (55.7)	466 (62.1)	
Alcohol drinking status				0.619
Current	465 (31.0)	232 (30.9)	233 (31.0)	
Former	91 (6.1)	50 (6.7)	41 (5.5)	
Never	946 (63.0)	469 (62.5)	477 (63.5)	
Hypertension	697 (46.4)	350 (46.6)	347 (46.2)	0.918
*PEMT* rs7946				0.101
TT	49 (3.3)	20 (2.7)	29 (3.9)	
CT	684 (45.5)	328 (43.7)	356 (47.4)	
CC	769 (51.2)	403 (53.7)	366 (48.7)	
Clinical measures				
BMI (kg/m^2^)	25.2 ± 3.6	25.0 ± 3.8	25.3 ± 3.4	0.110
Average SBP (mm Hg)	144.7 (132.0, 158.0)	143.3 (130.0, 158.3)	145.7 (133.3, 158.0)	0.203
Average DBP (mm Hg)	83.0 (75.7, 91.7)	82.7 (74.3, 92.0)	83.0 (76.0, 91.0)	0.398
Fasting glucose (mmol/L)	5.9 (5.4, 6.6)	6.0 (5.4, 6.6)	5.9 (5.5, 6.6)	0.595
Triglycerides (mmol/L)	1.2 (0.8, 1.8)	1.1 (0.8, 1.8)	1.2 (0.8, 1.9)	0.114
Total cholesterol (mmol/L)	6.1 (5.3, 7.0)	6.0 (5.2, 6.9)	6.1 (5.3, 7.0)	0.082
Serum concentrations for components of OCM				
Hcy (μmol/L)	12.6 (10.5, 15.4)	12.6 (10.3, 15.4)	12.7 (10.5, 15.4)	0.582
Vitamin B-12 (pg/mL)	515.8 (379.9, 679.9)	518.9 (382.8, 692.0)	510.1 (374.4, 659.8)	0.192
Total folate (ng/mL)	8.9 (6.2, 13.9)	9.1 (6.0, 13.9)	8.8 (6.4, 13.5)	0.965
Choline (ng/mL)	2.2 (1.8, 2.7)	2.2 (1.7, 2.7)	2.2 (1.8, 2.7)	0.433
Betaine (ng/mL)	6.1 (4.8, 7.7)	6.2 (4.9, 7.8)	5.9 (4.8, 7.4)	0.054
Choline-to-betaine ratio	0.361 (0.278, 0.470)	0.358 (0.269, 0.453)	0.366 (0.285, 0.490)	0.033

Abbreviations: DBP, diastolic blood pressure; Hcy, homocysteine; *PEMT*, phosphatidylethanolamine N-methyltransferase; SBP, systolic blood pressure; 5-MTHF, 5-methyltetrahydrofolate.

1 The summary statistics are presented *n* (%) for categorical variables and mean ± SD or median (quartile 1, quartile 3) for continuous variables.

### Association between *PEMT* rs7946 and risk of incident cancer

As shown in Table 2, after adjusting for potential confounders, CT and CC carriers conferred an increasing trend for digestive system cancer risk compared with participants with the TT genotype. Given the small number of TT carriers, we combined the 2 genotypes with the T allele (TT and CT) as the reference group. A substantially higher risk of digestive system cancer was seen for those with the CC genotypes (adjusted OR: 1.31; 95% CI: 1.04, 1.66; *P* = 0.023) of *PEMT* rs7946 in the conditional logistic regression model.

**TABLE 2:** Association between *PEMT* rs7946 genotypes and risk of digestive system cancer (pairs = 751)^1^

*PEMT* rs7946	*N*	Cases/controls	Crude model		Adjusted model	
			OR (95% CI)	*P* value	OR (95% CI)	*P* value
Individual genotypes						
TT	49	20/29	Reference		Reference	
CT	684	328/356	1.32 (0.74, 2.36)	0.344	1.49 (0.76, 2.91)	0.247
CC	769	403/366	1.60 (0.89, 2.86)	0.113	1.91 (0.97, 3.75)	0.061
*P*-trend				0.031		0.012
Combined genotypes						
TT/CT	733	348/385	Reference		Reference	
CC	769	403/366	1.23 (1.00, 1.52)	0.051	1.31 (1.04, 1.66)	0.023

Abbreviations: OR, odds ratio; *PEMT*, phosphatidylethanolamine N-methyltransferase.

1 Conditional logistic regression models were used. Adjusted for education level, smoking status, alcohol drinking status, average SBP, BMI, total cholesterol, triglycerides, fasting glucose, Hcy, vitamin B-12, total folate, choline, betaine, and the ratio of choline to betaine (choline:betaine).

### Stratification analyses

**FIGURE 2:**
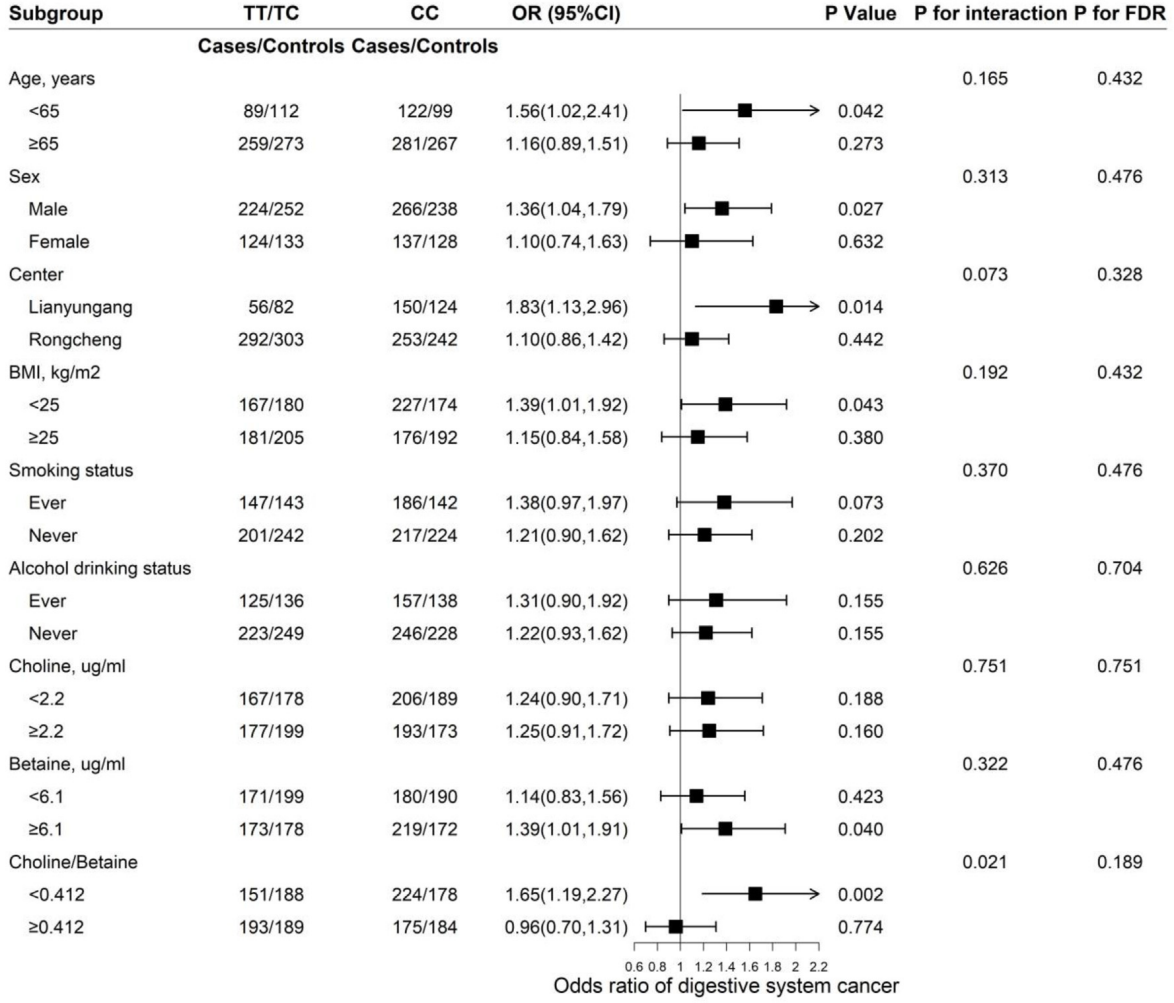
The association between *PEMT* rs7946 and incident risk of digestive system cancer in various subgroups. Unconditional logistic regression models were used. *P* values for interaction were calculated using log-likelihood ratio tests. Each subgroup analysis was adjusted, if not stratified, for age, sex, center, smoking status, alcohol drinking status, average SBP, BMI, total cholesterol, triglycerides, fasting glucose, Hcy, vitamin B-12, folate, choline, betaine, and choline-to-betaine ratio. *P* for FDR represents false discovery rate–adjusted *P*-interaction. OR, odds ratio; FDR, false discovery rate.

Stratified analyses were performed to assess the association of *PEMT* rs7946 with risk of digestive system cancer in various subgroups (Figure 2). None of the other variables, including age (<65 compared with ≥65 y; *P*-interaction = 0.165), sex (*P*-interaction = 0.313), center (Lianyungang compared with Rongcheng; *P*-interaction = 0.073), current smoking status (ever compared with never; *P*-interaction = 0.370), alcohol drinking status (ever compared with never; *P*-interaction = 0.626), choline (<2.2 compared with ≥2.2 μg/mL; *P*-interaction = 0.751), and betaine (<6.1 compared with ≥6.1 μg/mL; *P*-interaction = 0.322), significantly modified the association between *PEMT* rs7946 and digestive system cancer risk.

However, a more pronounced association was observed between this variant and risk of digestive system cancer in participants with a lower choline-to-betaine ratio (<0.412, adjusted OR: 1.65; 95% CI: 1.19, 2.27, compared with those with a ratio of ≥0.412, adjusted OR: 0.96; 95% CI: 0.70, 1.31; *P*-interaction = 0.021). Specifically, we applied false discovery rate corrections to account for multiple comparisons, whereas the false discovery rate–adjusted *P* value was 0.189.

### Mediation analysis

In this study population, we compared the choline and betaine concentrations between carriers with CC genotype and TT/CT genotypes. We found that the choline concentrations in CC genotype carriers were lower than those in TT/CT carriers, although this difference was not statistically significant (*P* = 0.080). However, betaine concentrations in the CC carriers were significantly higher than those in TT/CT carriers (*P* = 0.040), and the ratio of choline to betaine (choline:betaine) was significantly different between CC and TT/CT genotypes (*P* = 0.001) (Supplemental Table 2). To examine whether these metabolite concentrations and the choline-to-betaine ratio could be mediators of the association between *PEMT* rs7946 and risk of digestive system cancer, we conducted a mediation analysis using the nonparametric bootstrapping method and utilized the mediate function in the mediation package (package version: 4.5.0).

**FIGURE 3:**
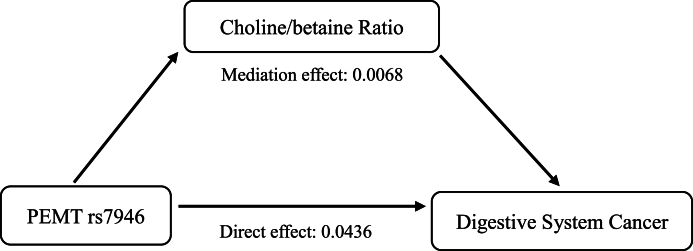
Choline-to-betaine ratio as a partial mediator on the association between variant *PEMT* rs7946 and risk of digestive system cancer (*P* = 0.018). The bootstrap test was used. The model adjusted for smoking status, alcohol drinking status, homocysteine, vitamin B-12, and total folate. *PEMT*, phosphatidylethanolamine N-methyltransferase.

A significant mediating effect was observed from the choline-to-betaine ratio (Supplemental Table 3 and Figure 3). Specifically, the result of the mediation analysis indicated that the choline-to-betaine ratio significantly mediated 13.55% of the association between *PEMT* rs7946 and digestive system cancer (*P* = 0.018).

## Discussion

To the best of our knowledge, this is the first study to estimate the association between *PEMT* rs7946 and risk of digestive system cancer among Chinese hypertensive adults. The following main findings were observed: *1*) CC carriers have 31% increased risk of digestive system cancer compared with the TT/CT combined genotypes (*P* = 0.023); *2*) this association was more pronounced in participants with a lower choline-to-betaine ratio (*P*-interaction = 0.021); and *3*) we proved a mediation effect of choline-to-betaine ratio in *PEMT* rs7946 and risk of digestive system cancer.

The enzyme *PEMT* catalyzes the reaction for de novo synthesis of choline, which is then oxidized to betaine, and betaine further serves as a substrate for methionine production, eventually becoming S-adenosylmethionine, the universal methyl donor for most methylation reactions, including DNA methylation [17], choline and betaine are key components in the OCM pathway, and they may be involved in DNA synthesis and repair owing to their close interrelationships and complex interactions with the folate cycle, thus potentially affecting tumor carcinogenesis.

Fundamental research using animal models has reported that rats fed a choline-deficient diet were much more likely to develop hepatocarcinoma [18,19], and a causal relationship between choline deficiency and carcinogenesis was proposed. In addition, it was reported that *PEMT* knockout mice had lower choline pools in the liver despite being fed sufficient amounts of dietary choline [20], implying that choline de novo biosynthesis from the *PEMT* pathway may be a predominate source of choline relative to dietary intake. However, a previous study found that the polymorphism of *PEMT* rs7946 is associated with changed enzyme activity [21], thus the association between this variant and cancer risk may be mediated by disturbed choline metabolism. In the Long Island Breast Cancer Study Project (LIBCSP) study, Xu et al. [13] examined the association between choline and breast cancer among Long Island females and found that breast cancer risk was reduced by 24% among females with high dietary intakes of choline.

Two studies have evaluated the associations between *PEMT* rs7946 and breast cancer. In the LIBCSP study, although neither *PEMT* rs7946 nor betaine intake were independently related to breast cancer risk, a significant interaction between this variant and dietary betaine intake was found (*P* < 0.05). Females carrying the CC genotype with the lowest betaine intake tertile had a 1.9-fold increased breast cancer risk [13]. Another case–control study showed *PEMT* rs7946 has no overall association with breast cancer, but a significant interaction was observed between choline intake and *PEMT* rs7946 (*P*-interaction = 0.029) in relation to breast cancer risk [12]. To our knowledge, this study is the first to examine the association between *PEMT* rs7946 and risk of digestive system cancer in a large, NCC study. We revealed that the T allele conferred a protective effect against digestive system cancer.

Stratified analyses were performed to assess the association between *PEMT* rs7946 and digestive system cancer risk in various subgroups. We introduced a parameter, the choline-to-betaine ratio, which was suggested as a better predictor of disturbed choline metabolism and more sensitive in association with cancer risk than either metabolite alone [22,23]. We noted a significant gene–environmental interaction from the choline-to-betaine ratio. In participants with a lower choline-to-betaine ratio (<0.412), a 65% increased risk among the CC genotype carriers (*P*-interaction = 0.021) was observed. This finding suggests that relatively sufficient serum choline concentrations may be an effective countermeasure against the increased cancer risk induced by the CC genotype of *PEMT* rs7946. Therefore, dietary intake containing choline and/or choline supplements may modify the susceptibilities to digestive system cancer of individuals with the CC genotype, but these results and inferences need to be replicated and confirmed carefully by large prospective studies in the future.

In addition, we hypothesized that *PEMT* rs7946 may disturb the concentrations of choline-related metabolites and, thus, influence risk of cancer in corresponding genotype carriers. In this study population, we found that compared with TT/CT combined genotypes, the CC genotypes, which showed a significantly increased risk of digestive system cancer (Table 2), tended to have relatively lower serum choline concentrations (*P* = 0.080), and significantly higher serum betaine concentrations (*P* = 0.040) (Supplemental Table 2). These divergent associations of serum choline and betaine are unexpected because betaine is derived from choline oxidation in vivo. However, given that this process may be modulated by 2 other enzymes, choline dehydrogenase and betaine aldehyde dehydrogenase, disturbed enzyme activities would be expected to result in different substrate concentrations. Additionally, betaine also serves as a source of methyl groups for the synthesis of PC through the reaction catalyzed by the enzyme *PEMT*; thus, this imbalance of choline and betaine is possible.

Given the significant association and the theoretical causality between the *PEMT* rs7946 and digestive system cancer, we hypothesized that this relationship may be mediated by the concentrations of choline-related metabolites. To test this hypothesis, a mediation analysis was conducted. Although we failed to confirm any mediating role of choline and betaine, we proved that the choline-to-betaine ratio partially mediated the association of *PEMT* rs7946 with digestive system cancer risk in this population (Supplemental Table 3 and Figure 3). To our knowledge, this is the first study to confirm the mediating effects of the choline-to-betaine ratio on the relationship between *PEMT* rs7946 and cancer risk. This finding will contribute to the understanding of the role of this variant in cancer susceptibility among Chinese adults and could be used as a basis for dietary/nutritional supplement regimens aimed at precise cancer prevention.

Of note, the variant *PEMT* rs7946 is associated with a valine-to-methionine (C-T) transition at amino acid position 175 (V175M). It has been reported that this base substitution may confer susceptibility to nonalcoholic fatty liver disease [21,24,25] and nonalcoholic steatohepatitis [26]. Another study conducted in a Chinese population found the T allele to be a risk factor for Alzheimer disease [27]. These data suggest that, in addition to risk of digestive system cancer, *PEMT* rs7946 also plays different roles in other diseases, making it noteworthy to illustrate its predictive value as a potential biomarker.

Several limitations should be noted. First, because data about pathologic character were lacking, we were unable to evaluate the relationship between *PEMT* rs7946 and subtypes of different digestive system cancers. Next, although we attempted to control for confounding and a broad set of covariates has been adjusted in the regression models, there is a potential for residual confounding by factors that were either not collected or not measured in this study. Third, this study was conducted among hypertensive Chinese adults with a specific composition of cancer subtypes (Supplemental Table 1), thus, the generalizability of our results to the general population should be evaluated with caution. Therefore, further studies are required to assess the validity of *PEMT* rs7946 as a reliable biomarker. This evaluation is crucial in establishing its potential as a risk marker for digestive system cancer.

In conclusion, this study provides evidence that supports a significant association between *PEMT* rs7946 and digestive system cancer risk, especially in individuals with a lower choline-to-betaine ratio. Our results also suggest that the choline-to-betaine ratio may partially mediate the association between *PEMT* rs7946 and digestive system cancer risk. These findings contribute to our understanding of the role of *PEMT* rs7946 in cancer susceptibility among Chinese adults and provide a potential basis for designing dietary/nutritional supplement regimens aimed at precise cancer prevention.

## Author contributions

The authors’ responsibilities were as follows – QH, HS, PQ: contributed to the study conception and design; PC, YS, BW, YW: contributed to the collection and assembly of the data; HZ, YW, QH: performed data analysis and interpretation; QH: drafted the manuscript; HS, PQ: performed manuscript review and editing; and all authors: reviewed and approved the final version of the manuscript.

## Conflict of interest

The authors declare that they have no competing interests.

## Funding

This project was supported by the National Key Research and Development Program, 2022YFC2009600 and 2022YFC2009601 (to HS); the Guangdong Provincial Key Laboratory of H-type Hypertension and Stroke Precision Prevention Research and Development Enterprise, 2020B121202010 (to PC); the Science, Technology and Innovation Committee of Shenzhen, JSGG20201103153807021 and KCXFZ20211020163801002 (to BW); the Department of Science and Technology of Guangdong Province, and the Development and Reform Commission of Shenzhen Municipality, XMHT20220104055 (to BW); the National Natural Science Foundation of China, 31970752 (to PQ); Science, Technology, Innovation Commission of Shenzhen Municipality, JCYJ20190809180003689, JSGG20200225150707332, JCYJ20220530143014032, ZDSYS20200820165400003 WDZC20200820173710001, WDZC20200821150704001 (to PQ); Shenzhen Bay Laboratory Open Funding, SZBL2020090501004 (to PQ); Department of Chemical Engineering-iBHE special cooperation joint fund project, DCE-iBHE-2022-3 (to PQ); Tsinghua Shenzhen International Graduate School Crossdisciplinary Research and Innovation Fund Research Plan, JC2022009 (to PQ); and Bureau of Planning, Land and Resources of Shenzhen Municipality, (2022) 207 (to PQ). The funder had no role in the study design, the data collection, analysis, and interpretation, or the manuscript writing.

## Data availability

Data described in the manuscript, code book, and analytic code will be made available on request pending application and approval.
