# Supplementary material for: The Mediating Effect of the Choline-to-Betaine Ratio on the Association Between PEMT rs7946 and Digestive System Cancer: A Nested Case–Control Study in a Chinese Population
Source: Curr Dev Nutr. 2024 Jan 4;8(2):102075. doi: 10.1016/j.cdnut.2024.102075 (PMC10862518; doi:10.1016/j.cdnut.2024.102075)
Supplement: Multimedia component 1 [file mmc1.docx]

# The mediation effect of choline/betaine ratio on the relationship between PEMT rs7946 and digestive system cancer among a Chinese population with hypertension

Qiangqiang He

**Supplemental Table 1. The subtypes of “digestive system cancers” included in this study and their respective quantities**

| ICD-10 code | Site of cancer occurrence | Number of cases |
| --- | --- | --- |
|  |  |  |
| C15 | Esophagus | 70 |
| C16 | Stomach | 200 |
| C17 | Small intestine | 11 |
| C18 | Colon | 84 |
| C19 | Rectosigmoid junction | 1 |
| C20 | Rectum | 129 |
| C21 | Anus and anal canal | 2 |
| C22 | Liver | 159 |
| C23 | Gallbladder | 4 |
| C24 | Other and unspecified parts of the biliary tract | 22 |
| C25 | Pancreas | 68 |
| C26 | Other and unspecified malignant neoplasms of digestive organs | 1 |

**Supplemental Table 2. The concentrations of serum choline, betaine, and choline/betaine ratio by different PEMT rs7946 genotypes (combined TT and CT genotype).**

| **Metabolites** | **Concentration**  **Median(Q1, Q3)** | **PEMT rs7946 genotype** | | **P-value** |
| --- | --- | --- | --- | --- |
|  |  | **TT/CT** | **CC** |  |
| N | 1502 | 733 | 769 |  |
| Choline | 2.2 (1.8, 2.7) | 2.2 (1.8, 2.7) | 2.1 (1.7, 2.7) | 0.080 |
| Betaine | 6.1 (4.8, 7.7) | 6.0 (4.7, 7.4) | 6.1 (4.9, 7.8) | 0.040 |
| Choline/Betaine | 0.361(0.278,0.470) | 0.375(0.292,0.488) | 0.353(0.268,0.455) | 0.001 |

For independent samples, the Mann-Whitney U test was used.

**Supplemental Table 3. Mediation analyses of serum choline, betaine, and choline/betaine ratio.**

|  | **Mediation effect** | | **Direct effect** | | **Total effect** | | **Mediation effect prop (%)** | |
| --- | --- | --- | --- | --- | --- | --- | --- | --- |
|  | Estimate | P-value | Estimate | P-value | Estimate | P-value | |  |
| Choline | 0.00250 | 0.392 | 0.04832 | 0.048 | 0.05082 | 0.040 | | 4.92 |
| Betaine | 0.00263 | 0.102 | 0.04775 | 0.052 | 0.05038 | 0.040 | | 5.22 |
| Choline/Betaine | 0.00683 | 0.018 | 0.04360 | 0.076 | 0.05043 | 0.038 | | 13.55 |

The bootstrap test was used.

Models adjusted for smoking status, alcohol drinking status, homocysteine, vitamin B12, and total folate.
